# Psychological benefits of pre-conceptional and pre-marital genetic diagnosis in conservative societies

**DOI:** 10.1192/j.eurpsy.2022.1525

**Published:** 2022-09-01

**Authors:** N. Bouayed Abdelmoula, B. Abdelmoula, S. Kammoun, F. Abid, S. Aloulou

**Affiliations:** Medical University of Sfax, Genomics Of Signalopathies At The Service Of Medicine, Sfax, Tunisia

**Keywords:** Genetic screening, conservative societies, pre-conceptional diagnosis, pre-marital diagnosis

## Abstract

**Introduction:**

Preconceptional genetic diagnosis help couples of genetic disorders carrier risk making an informed reproductive decision. The risk is considerably higher for consanguineous couples. Premarital screening can also offers a crucial health assessment of soon-to-be married couples with genetic risk factors based on specific family history. However, such approach is not usually easy to manage in conservative societies, particularly when the affected family refuse to deliver the necessary information about the genetic condition considered as a taboo.

**Objectives:**

Here, we addressed the psychological benefits of preconceptional and premarital genetic diagnosis through a retrospective study about the preconceptional diagnosis inquiries in our genetic counselling.

**Methods:**

In order to assess requests for autosomal recessive disorders during ten years of our genetic counselling activity at the medical university of Sfax, we reviewed 2500 medical files.

**Results:**

Three couples were recorded for genetic preconceptional diagnosis. Another couple was documented for seeking a premarital screening for an unknown neuropathy before wedding engagement decision. This single case was referred to us because of a familial history of a severe neuropathy that was noted in the offspring of a shared cousin. The couple was unable to bring us more information about the genetic condition because of the familial repugnance.

**Conclusions:**

Although our study is limited at the genetic level, it could be socially interesting because it showed the negative attitudes of the general population towards the genetic conditions and the familial responsiveness, as well as the reticence of physicians towards genetic preconceptional and premarital carrier diagnosis.

**Disclosure:**

No significant relationships.

